# Characteristics of the New Insulin-Resistant Zebrafish Model

**DOI:** 10.3390/ph14070642

**Published:** 2021-07-04

**Authors:** Youn Hee Nam, Isabel Rodriguez, Sung Woo Shin, Ji Heon Shim, Na Woo Kim, Min Cheol Kim, Seo Yule Jeong, Wanlapa Nuankaew, Bin Na Hong, Hyunggun Kim, Tong Ho Kang

**Affiliations:** 1 Department of Oriental Medicine Biotechnology, Kyung Hee University, Yongin 17104, Gyeonggi, Korea; 01030084217@hanmail.net (Y.H.N.); isabelula3r@gmail.com (I.R.); 01073205620@khu.ac.kr (S.W.S.); jee1015235@gmail.com (J.H.S.); nawoonifty@khu.ac.kr (N.W.K.); kminchel1213@gmail.com (M.C.K.); tjdbf26@gmail.com (S.Y.J.); wanlapa.nuankaew@gmail.com (W.N.); habina22@hanmail.net (B.N.H.); 2 Department of Biomechatronic Engineering, Sungkyunkwan University, Suwon 16419, Gyeonggi, Korea

**Keywords:** insulin resistance, zebrafish, pancreatic islet, MAPK signaling, calcium signaling

## Abstract

Insulin resistance, which occurs when insulin levels are sufficiently high over a prolonged period, causing the cells to fail to respond normally to the hormone. As a system for insulin resistance and diabetes drug development, insulin-resistant rodent models have been clearly established, but there is a limitation to high-throughput drug screening. Recently, zebrafish have been identified as an excellent system for drug discovery and identification of therapeutic targets, but studies on insulin resistance models have not been extensively performed. Therefore, we aimed to make a rapid insulin-resistant zebrafish model that complements the existing rodent models. To establish this model, zebrafish were treated with 10 μM insulin for 48 h. This model showed characteristics of insulin-resistant disease such as damaged pancreatic islets. Then we confirmed the recovery of the pancreatic islets after pioglitazone treatment. In addition, it was found that insulin-resistant drugs have as significant an effect in zebrafish as in humans, and these results proved the value of the zebrafish insulin resistance model for drug selection. In addition, RNA sequencing was performed to elucidate the mechanism involved. KEGG pathway enrichment analysis of differentially expressed genes showed that insulin resistance altered gene expression due to the MAPK signaling and calcium signaling pathways. This model demonstrates the utility of the zebrafish model for drug testing and drug discovery in insulin resistance and diabetes.

## 1. Introduction

Insulin resistance and pancreatic β-cell dysfunction characterize type 2 diabetes (T2D). Hyperinsulinemia can lead to insulin resistance by downregulating the mediators of the insulin signaling pathway [1]. In vivo studies exposing excessive insulin to rodent demonstrated insulin resistance, causing T2D [2,3,4]. Over time, islet β-cell compensation for insulin resistance fails, resulting in a progressive loss in β-cell function [5]. β-cell dysfunction is associated with the loss of β-cell mass due to apoptosis [6]. The time of the first occurrence of β-cell dysfunction is the beginning of T2D development. Inflammation, endoplasmic reticulum stress and mitochondrial-derived oxidative stress are involved in the development of insulin resistance [7,8,9,10]. 

It is well-identified that insulin is produced in the pancreatic islets by β-cells [11]. The mechanism involved in insulin secretion is regulated by the Ca^2+^ channel and ATP-mediated K^+^ channel in β-cells. The increase of intracellular ATP by glucose metabolism causes the closure of ATP-mediated K^+^ channels. As a result, a change of the potential of the cell membrane is generated, which opens a voltage-gated Ca^2+^ channel to increase the intracellular Ca^2+^ concentration. Then, increased intracellular Ca^2+^ concentration promotes insulin exocytosis from β-cells [12]. Conversely, ATP-mediated K^+^ channel opening induces hyperpolarization of the cell membrane that blocks Ca^2+^ channel openings and the intracellular influx of calcium, and thereby suppresses insulin secretion [13].

Insulin secretion, by the mechanism associated with the Ca^2+^ signaling pathway, acts on the tissue cells to promote intracellular glucose uptake. The mechanism of glucose uptake mediated by insulin is as follows. Insulin, secreted from β-cells through the Ca^2+^ signaling pathway, binds to tyrosine kinase receptors in tissue cells and activates the intracellular PI3K/AKT signaling pathway. Consequently, insulin promotes the expression of GLUT4 (Glucose Transporter Type 4) in cell membranes and increases the glucose uptake in tissue cells [14].

To investigate the multiple mechanisms, zebrafish (*Danio rerio*) are emerging as an animal model for interpreting the mechanisms underlying pathologies caused by transformed metabolism [15]. The zebrafish has been increasingly used to study diabetes and its related diseases thanks to its high similarities in organ physiology and metabolism compared to mammals [16,17,18,19]. The zebrafish is identified as an excellent system for the discovery and characterization of new diagnostic and therapeutic targets [20].

Glucose metabolism in both adult zebrafish and zebrafish embryos shares similarities with humans and other mammals [21,22]. PCR analysis revealed that carbohydrate-regulating genes of both adult and zebrafish larvae were identical with mammals. At 4 dpf, the pancreatic islets in zebrafish larvae are still immature but already functional. At that stage, organ development is completed through the fusion of the ventral anterior bud and dorsal posterior bud from the gut tube [23,24]. Moreover, a type 2 diabetes mellitus model of an adult zebrafish with automatic overfeeding was reported. This zebrafish model showed an increased level of fasting blood glucose, impaired glucose tolerance and improving effects of hyperglycemia when using anti-diabetic drugs: metformin and glimepiride [25]. Also, a hyperinsulinemia-derived insulin resistance model was reported. This model was developed using 4-dpf zebrafish by injection of high-dose human insulin, and the ptpn6 gene was suggested to be involved in hyperinsulinemia-derived insulin resistance and immune suppression [26]. Insulin resistance-induced zebrafish using glucose had an induction period of 30 days similar to that of mice [27]; such an advantage of a rapid model in zebrafish has not yet been explored. Therefore, we aim to make rapid insulin resistance by directly exposing excess insulin.

We established insulin resistance by hyperinsulinemia in a zebrafish model and confirmed the change of pancreatic islet using 2-(*N*-(7-nitrobenz-2-oxa-1,3-diazol-4-yl)amino)-2-deoxyglucose (2-NBDG) staining method, which has two functions: staining the pancreatic islet tissue and determining the glucose uptake. Fluorescent glucose bioprobes, including 2-NBDG, are designed in three parts: glucose, a fluorescent dye and a linker that connects both parts [28]. Of use, 2-NBDG allows us to visualize glucose uptake and is compatible with fluorescent microscopy [29]. Therefore, 2-NBDG treatment was used to stain the pancreatic islet tissue and determine the glucose uptake.

We verified the recovery of pancreatic islets and islet β-cells after pioglitazone treatment. RNA sequencing was performed to investigate this mechanism and the Kyoto Encyclopedia of Genes and Genomes (KEGG) pathway was evaluated via an enrichment analysis of differentially expressed genes (DEGs).

Then, we assessed the expression of an upregulated gene *map2k6* and downregulated genes *cacna1c* and *cacna1d* by insulin resistance. In this study, we demonstrated that insulin resistance altered gene expression due to the MAPK signaling and calcium signaling pathways.

## 2. Results

### 2.1. Short-Term Insulin Treatment

**Figure 1:**
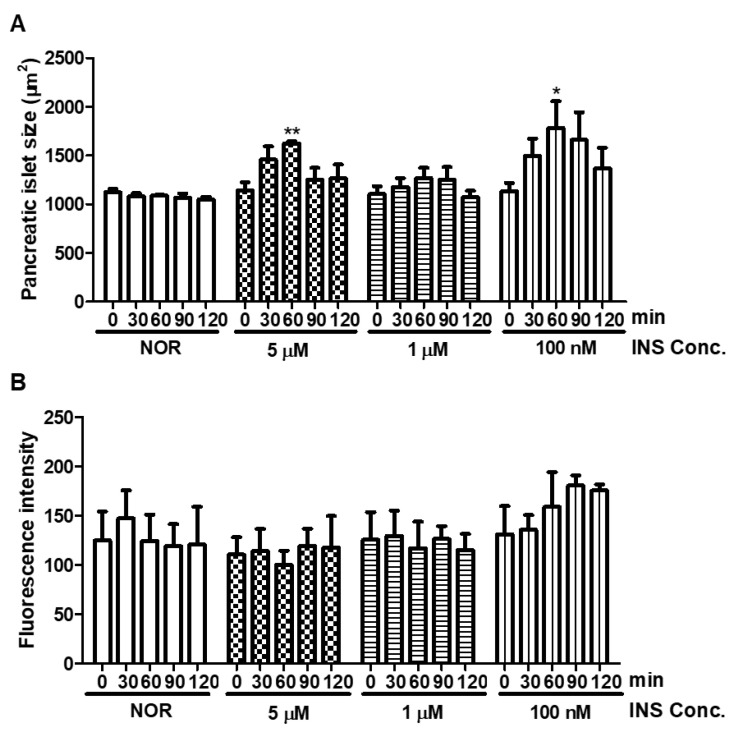
Changes in pancreatic islet following insulin (INS) treatment. (**A**) Dose- and time-dependent changes in pancreatic islet size caused by insulin were analyzed using Focus Lite software (*n* = 20). (*) *p* < 0.05 and (**) *p* < 0.01; compared to 0 min. (**B**) Fluorescence intensity of the pancreatic islet following insulin treatment.

The pancreatic islet of 5-dpf zebrafish treated insulin at 0.1, 1, and 5 μM in the short-term (0–120 min) were observed. According to a previous study, 100 nM of human insulin-microinjected zebrafish larvae showed ptpn6 expression stimulated by hyperinsulinemia [26]. In addition, hyperinsulinemia conditions led to pancreatic islet cell damage [5]. Therefore, we had expected that the pancreatic islet would be damaged after insulin treatment in zebrafish larvae. The normal group was used as the basis comparison with respect to induction of pancreatic islet damage from insulin by measuring the pancreatic islet size and fluorescence intensity. Our data showed a time-dependent tendency, with increasing pancreatic islet size at 60 min, then decreasing over time (Figure 1). 

### 2.2. Long-Term Insulin Treatment

**Figure 2:**
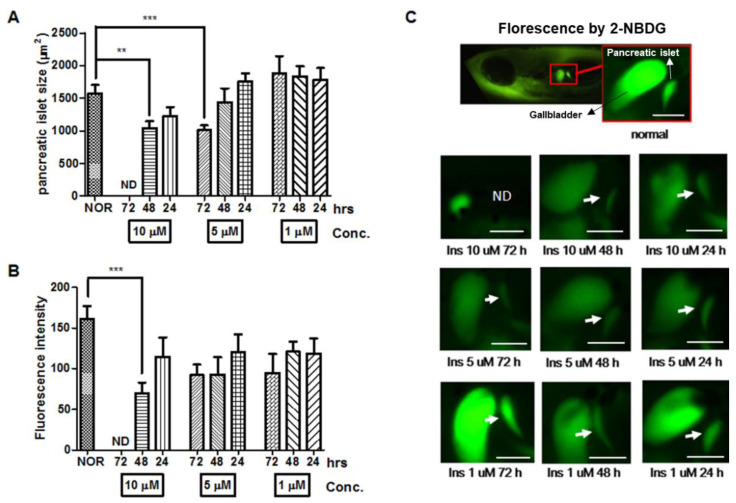
Dose- and time-dependent changes in pancreatic islet following insulin (INS) treatment. (**A**) Dose- and time-dependent changes in pancreatic islet size caused by insulin were analyzed using Focus Lite software (*n* = 20). (**) *p* < 0.01 and (***) *p* < 0.001; compared to normal group (NOR). (**B**) Fluorescence intensity of the pancreatic islet following insulin treatment using image J software. (***) *p* < 0.001; compared to NOR. (**C**) Fluorescent microscopic images of the pancreatic islet. The white arrow indicates the location of the pancreatic islet. Scale bar = 100 μm.

In order to study an insulin resistance model in zebrafish, high-dose insulin was given to treat zebrafish larvae for the long-term. During short-term treatment, the pancreatic islet did not change after insulin treatment for 30–120 min. In a previous study, a T2D model for long-term insulin exposure was established in mice [2]. Also, T2D patients generally undergo gradual changes of β-cell compensation, β-cell dysfunction and β-cell failure until it leads to insulin resistance. Thus, we hypothesized that long-term exposure to a high dose of insulin treatment could induce insulin resistance [5]. The pancreatic islet in zebrafish, as a result of high-dose insulin, can be damaged in a similar way to mammalian models. The average pancreatic islet size and fluorescence intensity of 7-dpf zebrafish were 1,575.25 ± 439.39 μm^2^ and 161.08 ± 68.04, respectively. After treatment with 10 μM insulin for 72 h, the treated zebrafish did not reveal the pancreatic islet, so it was not possible to measure the size and fluorescence of the pancreatic islet. Treatment with 10 μM insulin for 48 h significantly decreased the size (33.76%, *p* = 0.0049) and fluorescence intensity (56.63%, *p* = 0.0002) of the pancreatic islet compared to the normal group. Pancreatic islet size with a treatment of 10 μM insulin for 24 h did not significantly decrease compared to the normal group. Data showed that the zebrafish treated with 5 μM insulin for 24 h and 48 h showed a comparable pancreatic islet size to the normal group, while the zebrafish treated with 5 μM insulin for 72 h demonstrated a significantly reduced pancreatic islet size (34.32%, *p* = 0.0003). Treatment with 1 μM insulin led to an increased pancreatic islet size compared to the normal group (Figure 2).

To establish an insulin-resistant zebrafish model, 10 μM of insulin was selected as the optimal concentration for induction of pancreatic islet damage, and 48 h was selected as the optimal treatment time as 72 h treatment caused severe damage. Although 5 uM of insulin treatment for 72 h significantly reduced the pancreatic islet size, there was no significant decrease in fluorescence intensity.

### 2.3. Change of the Islet β-Cells with Excess Insulin Treatment

**Figure 3:**
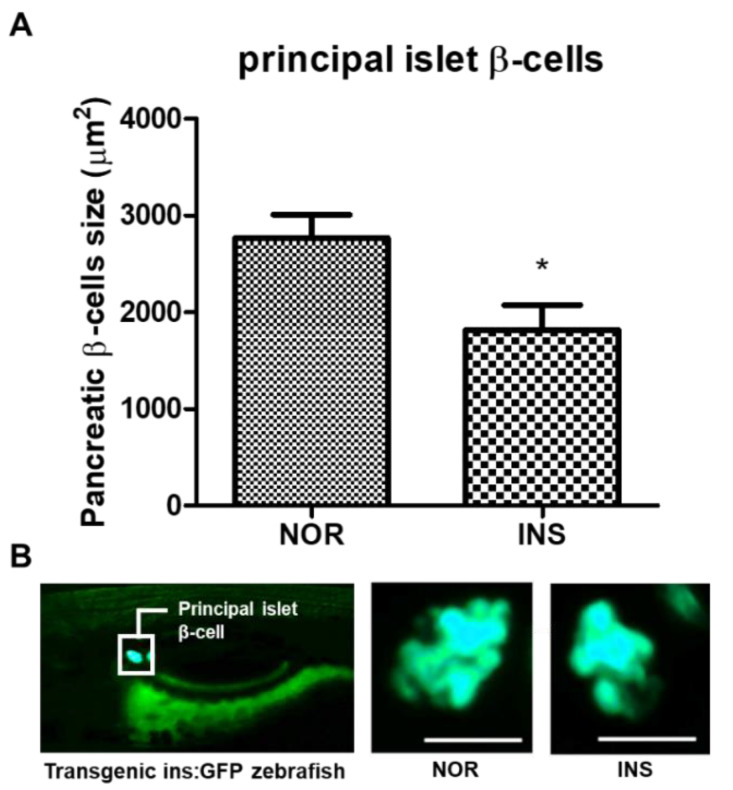
Changes in pancreatic β-cells following insulin (INS) treatment. (**A**) Principal islet β-cells’ size of the excess insulin-treated zebrafish (*n* = 20). (*) *p* < 0.05; compared to normal (NOR). (**B**) Fluorescent microscopic image of the principal islet β-cells. Scale bar = 100 μm.

The 2-NBDG treatment is useful to visualize the pancreatic islet tissue. However, a fluorescent protein of the transgenic zebrafish ins:Green Fluorescence Protein (GFP) specifically shows the β-cells in pancreatic islets.. With long-term insulin treatment (10 μM insulin for 48 h group), both the fluorescence intensity and the decreased pancreatic size were significant, as shown by 2-NBDG staining. Therefore, we exposed the transgenic zebrafish to the same conditions in order to confirm β-cell dysfunction, by measuring the fluorescence and size of the pancreatic β-cells, since β-cells account for about 70% of pancreatic islets and represent the specific site of insulin secretion [30]. The change of the islets’ β-cells was observed with exposure to excess insulin. The pancreatic β-cells’ size in the insulin-treated group significantly decreased by 40.1% (*p* = 0.0177) compared to the normal group (Figure 3A).

### 2.4. Glucose Tolerance

**Figure 4:**
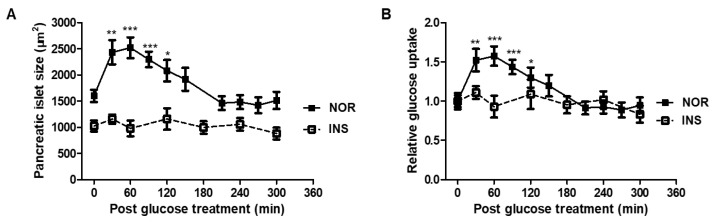
Glucose tolerance test (GTT) in insulin-treated zebrafish. (**A**) Size of pancreatic islet by glucose treatment (*n* = 20). (*) *p* < 0.05, (**) *p* < 0.01 and (***) *p* < 0.001; compared to 0 min. (**B**) Relative glucose uptake by glucose treatment (*n* = 20). (*) *p* < 0.05, (**) *p* < 0.01 and (***) *p* < 0.001; compared to 0 min.

A glucose tolerance test was performed to confirm the time required for the zebrafish to return the glucose levels to homeostasis. In the normal group, the pancreatic islet size and glucose level increased up to 60 min and then slowly decreased. At approximately 210 min, they maintained homeostasis. However, in the case of the insulin-treated group, glucose uptake did not significantly change (Figure 4).

Glucose uptake was evaluated through 2-NBDG from 0 to 300 min after exposure to 10 mM glucose for 20 min; 2-NBDG is a fluorescent dye that allows live observation of glucose uptake. In the normal group, the size and fluorescence intensity of the pancreatic islet increased up to 60 min and then decreased slowly. It was confirmed that the absorption of glucose up to 120 min was significantly higher than at 0 min. From 210 min, it was confirmed that the size and fluorescence intensity were maintained, similar to at 0 min. On the other hand, in the insulin-treated zebrafish, there were no changes in size and fluorescence intensity even after glucose exposure.

### 2.5. Insulin Sensitivity

**Figure 5:**
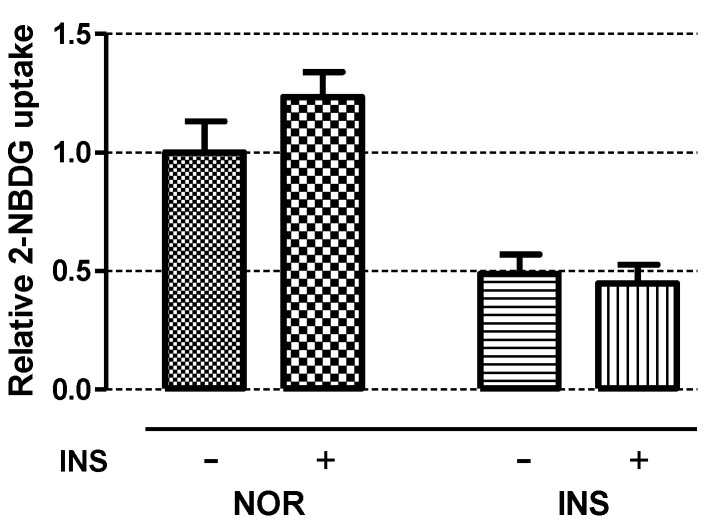
Insulin sensitivity test. 2-NBDG was used for measuring relative glucose uptake after treatment of 1 μM insulin for 1 h (*n* = 20). “+” means 1 μM insulin is treated for 1 h and “-” means non-treated in both NOR and INS group as control for comparing 2-NBDG uptake.

To evaluate the insulin sensitivity of insulin-treated zebrafish, normal and insulin-treated zebrafish larvae were treated with 1 μM insulin for 1 h. While the pancreatic islet size of the normal zebrafish significantly increased by 26.15% (*p* = 0.0434), the pancreatic islet size of the insulin-treated zebrafish did not increase after the small amount of insulin treatment (Figure 5).

### 2.6. Efficacy of Glimepiride (GLM), ile-pro-ile (IPI) and Acarbose (ABS) on Insulin-Treated Zebrafish Larvae

**Figure 6:**
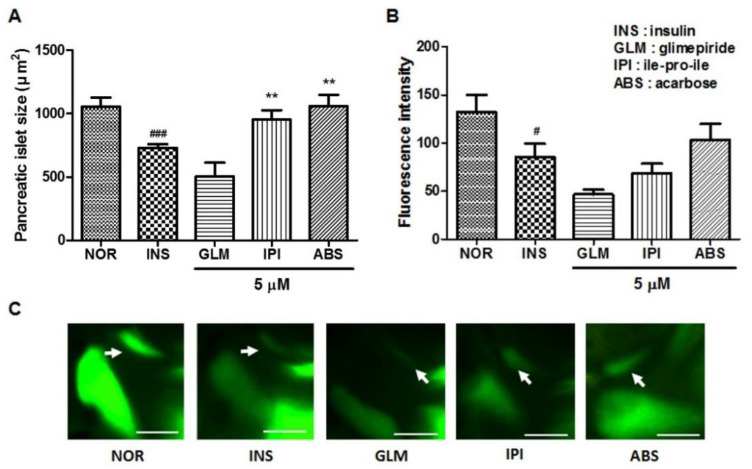
Effects of glimepiride (GLM), ile-pro-ile (IPI) and acarbose (ABS) on excess insulin-treated zebrafish. (**A**) Pancreatic islets’ size. (###) *p* < 0.001; compared to normal group (NOR). (**) *p* < 0.01; compared to insulin-treated group (INS). (**B**) Fluorescence intensity in the pancreatic islets. (#) *p* < 0.05; compared to NOR. (**C**) Representative pancreatic islet images of the NOR, INS, GLM-, IPI- and ABS-treated zebrafish. Pancreatic islets were stained using 2-NBDG. The white arrow indicates the location of the pancreatic islet. Scale bar = 100 μm.

To study the response of insulin-treated zebrafish larvae to anti-diabetes drugs, we evaluated the pancreatic islet with glimepiride (GLM), ile-pro-ile (IPI) and acarbose (ABS) treatment. The GLM-treated groups showed decreased pancreatic islet size and fluorescence intensity compared to the insulin-treated group. The IPI- and ABS-treated groups showed significantly increased pancreatic islet size (30.61%, *p* = 0.0020 and 44.81%, *p* = 0.0002, respectively) compared to the insulin-treated group. Fluorescence intensity showed a similar tendency in terms of pancreatic islet change (Figure 6).

### 2.7. Efficacy of Pioglitazone (PIO) on Insulin-Treated Zebrafish Larvae

**Figure 7:**
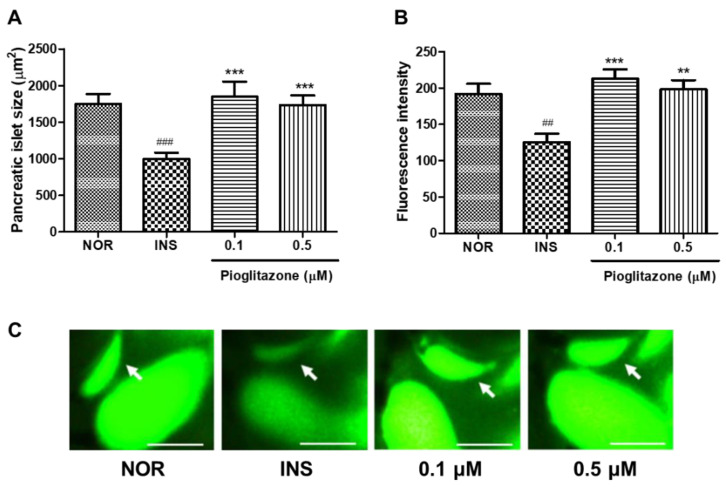
Effects of pioglitazone (PIO) on excess insulin-treated zebrafish. (**A**) Pancreatic islets’ size. (###) *p* < 0.001; compared to normal group (NOR). (***) *p* < 0.001; compared to insulin-treated group (INS). (**B**) Fluorescence intensity in the pancreatic islets. (##) *p* < 0.01; compared to NOR. (**) *p* < 0.01, (***) *p* < 0.001; compared to INS. (**C**) Representative pancreatic islet images of the NOR, INS, PIO-treated zebrafish. Pancreatic islets were stained using 2-NBDG. The white arrow indicates the location of pancreatic islet. Scale bar = 100 μm.

To study the response of insulin-treated zebrafish larvae to anti-diabetes drugs, we evaluated the pancreatic islet with pioglitazone (PIO) treatment. The 0.1 and 0.5 μM PIO-treated groups had significantly increased pancreatic islet size (51.61%, *p* = 0.0003 and 52.85%, *p* < 0.0001, respectively) compared to the insulin-treated group. Fluorescence intensity showed a similar tendency in terms of pancreatic islet change (Figure 7).

### 2.8. Free Glucose Level in Insulin-Treated Zebrafish Larvae

**Figure 8:**
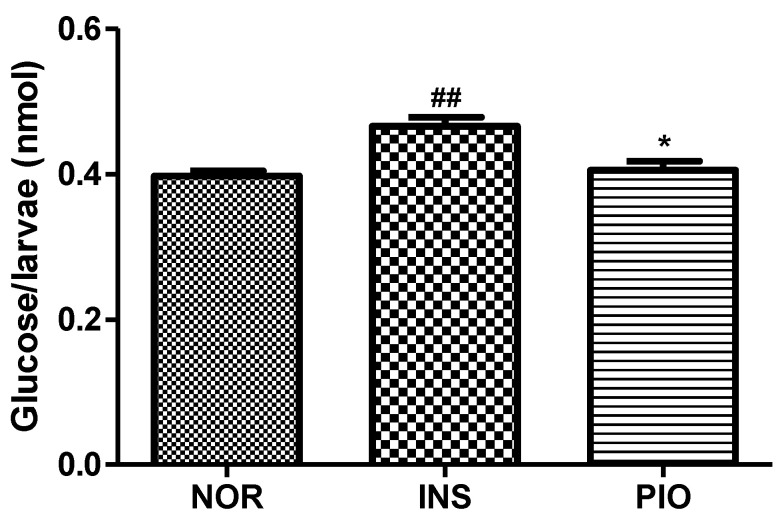
Free glucose level following pioglitazone (PIO) treatment in insulin-treated zebrafish. (##) *p* < 0.01; compared to normal (NOR). (*) *p* < 0.05; compared to insulin-treated group (INS).

To study the response of insulin-treated zebrafish larvae to anti-diabetes drugs, we evaluated the free glucose level with pioglitazone (PIO) treatment. The 0.1 μM PIO-treated groups showed a significantly decreased free glucose level (13.06%, *p* = 0.0128) compared to the insulin-treated group (Figure 8).

### 2.9. Differential Gene Expression Induced by Insulin-Treated Zebrafish

**Figure 9:**
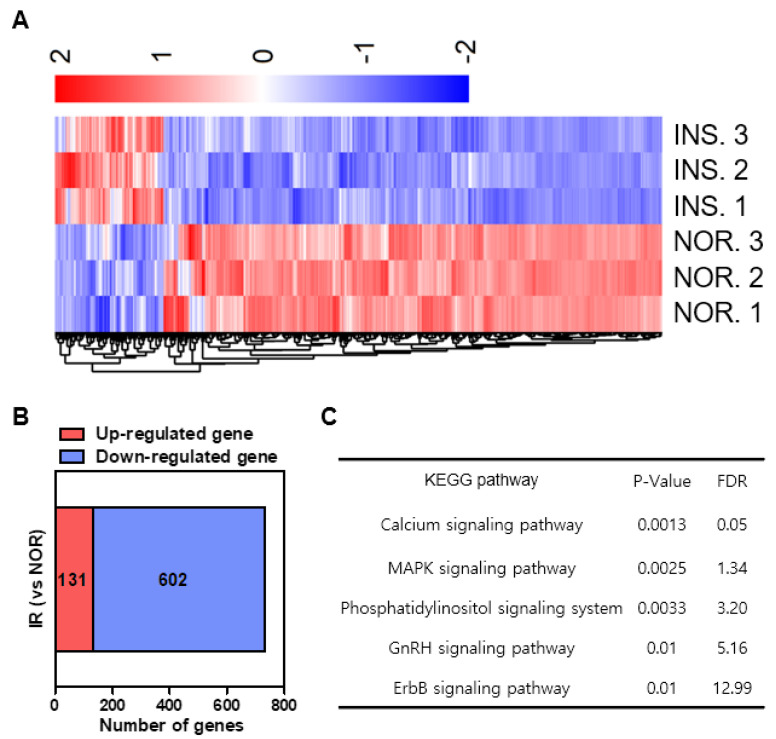
Differential gene expression induced by insulin-treated group (INS) compared to normal group (NOR). (**A**) Heat map based on RNA-sequencing analysis of the gene expression in zebrafish. (**B**) RNA-sequencing for the excess insulin treatment-regulated gene set. (**C**) KEGG pathways.

To investigate the mechanism of insulin resistance-induced damage, we identified the genes affected by excess insulin treatment using transcriptome analysis. RNA sequencing was performed to monitor differential gene expression in the genome of the excess insulin-treated zebrafish. Given the 30,805 genes expressed in zebrafish, 733 genes were differentially expressed (FDR <0.05, $\left|\log_{2}FC\right|$>1) by excess insulin treatment and the results are presented as a heat map in Figure 9A. Among these 733 genes, 131 were up-regulated and 602 genes were downregulated by insulin resistance (Figure 9B).

Next, KEGG analysis was performed to identify the functional pathways of these differentially expressed genes (Figure 9C). The expression of the *map2k6* gene was increased by a high dose of insulin treatment. The MAPK signaling (*map2k6*) was changed, indicating that the stress response and cell fate-related functions were changed by a high dose of insulin treatment. In addition, the expression of the *cacna1c* and *cacna1d* genes decreased with a high dose of insulin treatment. The calcium signaling could be an upstream pathway of the cascades.

### 2.10. Effect of PIO in Altered Gene Expression on Insulin-Treated Zebrafish

**Figure 10:**
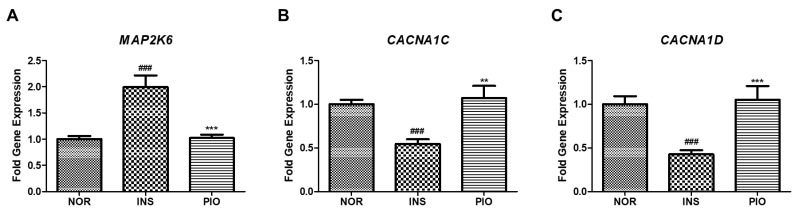
Differential gene expression induced by insulin-treated zebrafish. (**A**) The *map2k6* gene was determined by RT-qPCR. (###) *p* < 0.001; compared to normal group (NOR). (***) *p* < 0.001; compared to insulin-treated group (INS). (**B**) The *cacna1c* gene was determined by RT-qPCR. (###) *p* < 0.001; compared to normal group (NOR). (**) *p* < 0.01; compared to insulin-treated group (INS). (**C**) The *cacna1d* gene was determined by RT-qPCR. (###) *p* < 0.001; compared to normal group (NOR). (***) *p* < 0.001; compared to insulin-treated group (INS).

We examined the effect of PIO on the expression of the genes affected by excess insulin treatment as identified by RNA sequencing. When insulin-treated zebrafish were treated with PIO, the increased gene expression by insulin treatment, which is related to MAPK signaling (*map2k6*), was significantly downregulated (Figure 10A). In contrast, the expression of genes in pathways related to calcium signaling (*cacna1c* and *cacna1d*), which were all reduced by excess insulin treatment, was significantly rescued by PIO treatment (Figure 10B,C).

## 3. Discussion

In this study, we developed an insulin-resistant zebrafish model by excess insulin-induced insulin resistance. Hyperinsulinemia is known to lead to insulin resistance in mammals by downregulating the mediators of the insulin signaling pathway [1]. Insulin resistance causes loss of pancreatic islets and β--cell damage, and leads to diabetes.

To establish an insulin-resistant zebrafish model, we used a newly developed method to expose zebrafish to 10 μM of insulin for 48 h. After excess insulin treatment, the pancreatic islet of zebrafish was identified by 2-NBDG staining. Compared to the method using GFP-transgenic-line zebrafish, using 2-NBDG has the advantage that anyone can visualize zebrafish’s pancreatic islets if they have experimental protocols and reagents, without specific equipment and technological skills being needed.

However, 2-NBDG can also visualize the pancreatic islets as well as show a similar fluorescence with zebrafish expressing GFP. With the 2-NBDG staining method, the decreased size of the pancreatic islet means β-cell damage and fluorescence intensity is related to the effect of 2-NBDG uptake on the pancreatic islet. Fluorescence intensity is only measured on the pancreatic region attributively. Insulin is secreted in pancreatic islets’ β-cells and insulin resistance or hyperinsulinemia destroys β-cells via apoptosis, which has been previously reported as a decrease in the function and size of β-cells. Therefore, the criterion of insulin resistance was judged as a decrease in the pancreatic islet size of zebrafish. Excess insulin-exposed zebrafish showed serious damage in the pancreatic islets and β-cells. Before establishing this model, we developed a protocol to have zebrafish exposed to lower than 10 μM insulin for a short time. The pancreatic islet size increased by glucose uptake and then returned to normal. Therefore, we changed the protocol such that 3-dpf zebrafish larvae were exposed to high-concentration insulin for a longer time. So, 3-dpf zebrafish larvae were used in all experiments except the study of insulin with short-term exposure. It is reported that zebrafish larvae consume the nutrients stored in their yolk by 8 dpf. After a period of 8 dpf, they lack nutrients in their yolk so they need to consume nutrients from the external environment for survival [31].

However, the externally supplied glucose creates unpredictable variables for changes in blood glucose and insulin resistance in the zebrafish model, and the absolute amount of nutrients consumed by each individual cannot be the same. Therefore, to make a zebrafish model that only uses nutrients stored in their yolk, 3-dpf zebrafish larvae are used in long-term insulin treatment considering a total period of 96 h for zebrafish insulin-resistant model establishment. When high-dose insulin was administered to zebrafish, the pancreatic islet disappeared after exposure to 10 μM insulin for 72 h. Ultimately, we have found the optimal treatment time and dose for insulin resistance. In our zebrafish insulin resistance model, insulin resistance gradually reduced the pancreatic islet. Furthermore, we verified the pancreatic islets’ damage by 2-NBDG staining, and β-cell damage through the transgenic zebrafish model.

In an early stage in the progression of T2D, a period of normal and near-normal glycemia is observed for the reason of compensation of pancreatic β-cells by hypersecretion of insulin [32], and the pancreas tries to compensate for insulin resistance by increasing β-cell mass [33]. However, this period of β-cell compensation leads to β-cell failure with secretion of insufficient insulin, and diabetes ensues [32]. Referring to former studies, diabetes-induced C57BL/6 mice administered an insulin reagent (glargine) once a day for 8 weeks showed reduced pancreatic islet size [2]. We used zebrafish to shorten the induction time and confirmed decreased pancreatic islet size and glucose uptake in the live state. 

In the glucose tolerance test, glucose uptake of the normal group increased by 1.5-fold, but glucose uptake of the insulin resistance group did not change with the glucose treatment. Simultaneously, insulin-stimulated glucose uptake did not respond in the insulin-resistant group. Insulin resistance in the muscle and fat cells reduces glucose uptake [34]. These results suggest that insulin-stimulated glucose uptake is impaired in the zebrafish model.

The normal zebrafish showed a sensitivity response to the insulin treatment as an increased pancreatic islet, but the insulin-resistant zebrafish did not show a sensitivity reaction, with no change to the pancreatic islet.

Pioglitazone (PIO), of the thiazolidinedione class, is an agonist of peroxisome proliferator-activated receptors (PPARs) and improves hyperglycemia, reduces hyperinsulinemia and enhances β-cell function in a variety of insulin-resistant animal models [35,36]. In our study, the PIO-treated insulin resistance group demonstrated an improvement of damaged pancreatic islets. 

In addition, several anti-diabetes drugs were evaluated in injured pancreatic islets and the effects of ile-pro-ile as a DPP4 inhibitor, and acarbose as an α-glucosidase inhibitor were confirmed. ile-pro-ile (IPI) is Diprotin A classified as a dipeptidyl peptidase IV (DPP-IV) inhibitor [37]. The DPP-IV inhibitor inhibits the degradation of glucagon-like peptide-1 (GLP-1), related to insulin secretion and glucose tolerance [38]. Acarbose is classified as an α-glucosidase inhibitor and reduces the absorption of dietary glucose [39].

The glucose levels of insulin-treated zebrafish remained higher than those of normal zebrafish. These zebrafish showed, consistent with other models of insulin resistance, higher levels of plasma glucose [40]. Furthermore, PIO-treated zebrafish showed decreased glucose levels, consistent with existing rodent models [41].

RNA sequencing was performed to monitor the differential expression of genes by induced insulin resistance and identify the mode of action by insulin resistance. The upregulated genes by insulin resistance were enriched in the MAPK signaling pathway. It is well-established that MAPK pathway activation increases in type 2 diabetic patients and that p38-MAPK-related genes have an important role in the type 2 diabetes pathogenesis [42]. A member of the protein kinase family like a mitogen-activated protein (MAP) kinase is encoded by the *map2k6* gene. The phosphorylation and activation of the p38 MAP kinase related with inflammatory cytokines or environmental stress are essential components of the p38 MAP kinase-mediated signal transduction pathway [43]. In our model, the expression of the *map2k6* gene increased with insulin resistance. The *map2k6* gene is involved in phosphorylation and activation of the p38 MAP kinase, a serine/threonine-protein kinase, and p38 MAPK is related to cellular responses to inflammatory cytokines or environmental stress, including insulin resistance [44]. Activation of p38 MAPK leads to high levels of glucose, plasma free fatty acids (FFAs), inflammatory cytokines and overactivation of the cardiovascular renin-angiotensin system (RAS) in insulin resistance and T2DM [45]. In contrast, the expression of the *cacna1c* and *cacna1d* genes is decreased by insulin resistance. Insulin secretion needs the presence and activity of the L-type calcium channels, making them critical for insulin release. Decreased expression of calcium channel-related genes such as *cacna1c* and *cacna1d* is one of the characteristics of type 2 diabetes [46]. Calcium ions involved in the bidirectional communication of organelles and impaired communication are associated with insulin resistance and diabetes [47]. In summary, according to the results of RNA-sequencing analysis, the most affected genes in the KEGG analysis were the calcium signaling pathway and MAPK signaling pathway. Also, regulation of voltage-gated calcium channel activity and positive regulation of MAP kinase activity are the common gene-sets in the T2DM of both humans and zebrafish in gene-set enrichment analysis [25]. The *cacna1c* and *cacna1d* genes in the calcium signaling pathway and *map2k6* gene in the MAPK signaling pathway showed a greater difference in gene expression among the pathways in zebrafish. However, since those genes are a part of the genes associated with these pathways, further studies such as gene validation are required in the insulin-resistant zebrafish model regarding the calcium and MAPK signaling pathways.

In this study, we confirmed a decrease in size in pancreatic islets in zebrafish due to insulin exposure. This is a typical symptom seen in insulin-resistant animal models and diabetic patients, and may be useful as a model for drug testing and drug discovery of insulin resistance. We evaluated the pancreatic islet recovery and gene expression profiles of insulin-exposed zebrafish and demonstrated that they are suitable as an insulin-resistant zebrafish model.

## 4. Materials and Methods

### 4.1. Reagents and Equipment

Insulin Human Recombinant, D-glucose, Pioglitazone, ile-pro-ile, acarbose tricaine methanesulfonate and sea salts were purchased from Sigma Chemical Co. (St. Louis, MO, USA). 2-(*N*-(7-nitrobenz-2-oxa-1,3-diazol-4-yl)amino)-2-deoxyglucose (2-NBDG) was purchased from Invitrogen (Carlsbad, CA, USA). Glimepiride was purchased from Cayman Chemical Co. (Ann Arbor, MI, USA). Fluorescence microscopy was performed using an Olympus 1 × 70 microscope (Tokyo, Japan). For image analysis, Focus Lite (Focus Co, Gwangmyeong-si, Korea) and Image J (V 1.50i, National Institutes of Health, Bethesda, MD, USA) were used.

### 4.2. Zebrafish Maintenance and Embryo Collection

Wild-type AB strain zebrafish (*Danio rerio*) were maintained at the zebrafish system S type (1500 (W) × 400 (D) × 2050 (H) mm) (Genomic Design Bioengineering Co., Daejeon, Korea). Two pairs of zebrafish were placed in the spawning box overnight. The next day, the zebrafish started spawning after a period of light exposure for 30 min. Zebrafish embryos were collected at 3 h post-fertilization (hpf) for experiments and incubated in 0.03% sea salt solution in a petri dish. The embryos were maintained in a cycle of 14 h light: 10 h dark at 28.5 °C in an incubator.

### 4.3. Short-Term Insulin Treatment

Five-day post-fertilization (dpf) wild-type zebrafish were placed into six-well plates. First, the zebrafish larvae were treated with various concentrations of insulin (0, 0.1, 1 and 5 μM) over various time periods (0, 30, 60, 90 and 120 min). Following treatment, to obtain pancreatic islet images, the zebrafish larvae were stained with 40 μM 2-NBDG for 30 min and rinsed with 0.03% sea salt solution for 20 min. After treatment with 40 μM 2-NBDG, the zebrafish was laid on its side after anesthesia using 0.004% tricaine solution and mounted in the same position with only the left eye to be seen before capturing the pancreatic islets. Images were captured using a fluorescence microscope and analyzed using Focus Lite and Image J.

### 4.4. Long-Term Insulin Treatment

Three-day post-fertilization (dpf) wild-type zebrafish were placed into six-well plates. First, the zebrafish larvae were treated with various concentrations of insulin (0, 1, 5 and 10 μM) for various periods of time (24, 48 and 72 h). Following treatment to obtain pancreatic islet images, the zebrafish larvae were stained using 40 μM 2-NBDG for 30 min and rinsed with 0.03% sea salt solution for 20 min. After treatment with 40 μM 2-NBDG, the zebrafish was laid on its side after anesthesia using 0.004% tricaine solution and mounted in the same position with only the left eye to be seen before capturing the pancreatic islets. Images were captured by fluorescence microscopy and analyzed using Focus Lite and Image J software.

### 4.5. Change of Islet β-Cells by Excess Insulin Treatment

Transgenic zebrafish lines expressing GFP specifically in β-cells were obtained from the Zebrafish Organogenesis Mutant Bank (ZOMB). The GFP-tagged line was ins:GFP, which expresses GFP in the pancreatic islets [48]. Three-dpf ins:GFP zebrafish were treated with 0 or 10 μM insulin for 48 h in six-well plates. Following treatment, the zebrafish were exposed to 0.03% sea salt solution for 48 h and then the captured β-cells were analyzed using Focus Lite.

### 4.6. Glucose Tolerance Test

D-glucose was dissolved in 0.03% sea salt solution. The normal group and excess insulin-treated group were exposed to 10 mM glucose solution for 20 min. Following glucose treatment, pancreatic islets were observed after 0, 30, 60, 90, 120, 150, 180, 210, 240, 270 and 300 min.

### 4.7. Insulin Sensitivity

Seven-dpf normal and excess insulin-treated zebrafish larvae were placed onto 96-well plates. Normal and insulin-treated zebrafish larvae were treated with 1 μM insulin for 1 h and rinsed three times with 0.03% sea salt solution. After treatment, the zebrafish larvae were stained with 40 μM 2-NBDG for 30 min and rinsed three times with 0.03% sea salt solution for 20 min. The zebrafish was laid on its side after anesthesia using 0.004% tricaine solution and mounted in the same position with only the left eye to be seen before capturing the pancreatic islets. Images were captured by fluorescence microscopy and analyzed using Focus Lite and Image J.

### 4.8. Treatment of Glimepiride (GLM), ile-pro-ile (IPI), Acarbose (ABS), Pioglitazone (PIO) on Excess Insulin-Treated Zebrafish Larvae

Three-dpf zebrafish were placed into six-well plates. The zebrafish larvae were treated with 10 μM insulin for 48 h, rinsed three times with 0.03% sea salt solution and then treated with 5 μM GLM, IPI, ABS and PIO for 48 h. Following treatment, the zebrafish larvae were rinsed three times, stained with 40 μM 2-NBDG for 30 min and rinsed three times with 0.03% sea salt solution for 20 min. The zebrafish was laid on its side after anesthesia using 0.004% tricaine solution and mounted in the same position with only the left eye to be seen before capturing the pancreatic islets. Images were captured by fluorescence microscopy and analyzed using Focus Lite and Image J.

### 4.9. Free Glucose Assay

The free glucose of zebrafish larvae was determined using the Glucose Colorimetric/Fluorometric Assay Kit (Sigma-aldrich, St. Louis, MO, USA). Ten larvae were homogenized in 100 μL of glucose assay buffer. The homogenate was cleared by centrifugation and 10 μL of supernatant (equivalent of one larva) was measured [49]. The reaction was incubated for 30 min at 37 °C according to the manufacturer’s instructions. The optical density was measured at 570 nm in a Microplate Reader (PerkinElmer, Waltham, MA, USA)

### 4.10. mRNA Sequencing and Pathway Analysis

Three-dpf zebrafish were placed into six-well plates. The zebrafish larvae were treated with 10 μM insulin for 48 h and rinsed with 0.03% sea salt solution for 48 h. Following treatment, the zebrafish larvae were washed with PBS. Total RNA was extracted using TRIzol RNA Isolation Reagent (Invitrogen, Carlsbad, CA, USA) and then further purified using an RNeasy mini kit (QIAGEN, Hilden, Germany) to remove genomic DNA. To evaluate total RNA quantity and quality, an Agilent 2100 bioanalyzer (Agilent, Santa Clara, CA, USA) was used. Sequencing libraries were prepared by processing the isolated total RNA using the TruSeq Stranded mRNA Sample Preparation Kit (Illumina, San Diego, CA, USA). An Agilent 2100 Bioanalyzer (Agilent, Santa Clara, CA, USA) was used to evaluate the quality and size of the whole library. All libraries were quantified by qPCR using a CFX96 real-time system (Bio-Rad, Hercules, CA, USA) and sequenced on a NextSeq500 sequencer (Agilent, Santa Clara, CA, USA) with paired-end 75-bp and single 8-bp index read runs. The raw data of RNA analysis were converted into sequence data and then stored in a different format as a FASTQ file. Genes representing absolute fold change (FC) greater than 1.4 and *p* < 0.05 were differentially expressed between the groups. The datasets of significantly differential expression were processed as an aspect of exploiting the KEGG expression database. EnrichR was used for WikiPathways analysis [50].

### 4.11. Real Time-qPCR

The zebrafish were treated with 10 μM insulin for 48 h and then treated for 48 h with 0.1 μM PIO. After treatment, total RNA was extracted from zebrafish larvae with TRIzol™ reagent (Thermo Fisher Scientific Korea Ltd., Seoul, Korea) according to the manufacturer’s protocol. The relative mRNA expression level was measured by qPCR and β-actin was used to normalize mRNA expression. Then, 1 μg of total RNA was reverse-transcribed with Rever Aid First Strand cDNA Synthesis Kit (Thermo Fisher Scientific Korea Ltd., Seoul, Korea) according to the manufacturer´s protocol. The qPCR was performed in 10 μL reactions containing 5 μL of SYBR Select Master Mix (Applied-byosystems, Thermo Fisher Scientific Korea Ltd., Seoul, Korea), 1 μL of CDNA template, 1 μL of forward primer (10 pmol), 1 μL of reverse primer (10 pmol) and 2 μL of RNAase free water. The qPCR parameters were as follows: initial denaturation at 95 °C for 5 min, followed by 45 cycles of 95 °C for 15 s, 60 °C for 15 s and 72 °C for 20 s, and then, melting at 73 °C for 5 min. The expression of genes was analyzed by the $2^{-\Delta \Delta Ct}$ method. Primers sequences are listed in Table 1.

### 4.12. Statistical Analysis

Statistical analyses were performed using GraphPad Prism (version 5). Data were expressed as mean ± standard error of the mean (SEM). Statistical significance was determined using the repeated one-way ANOVA followed by Tukey’s test. The probability level for statistical significance was set at *p* < 0.05.

## 5. Conclusions

In conclusion, we demonstrated that the zebrafish model was suitable for assessing antidiabetic candidates by evaluation of the pancreatic islet recovery and gene expression profiles. This model demonstrates the utility of the zebrafish model for drug testing and drug discovery in insulin resistance and diabetes.

## Figures and Tables

**Table 1:** Primers for qPCR.

Gene Name	Forward Sequence	Reverse Sequence
β-Actin	CGA GCA GGA GAT GGG AAC C	CAA CGG AAA CGC TCA TTG C
*map2k6*	CCA CAG CAA TCT GTC AGT C	CTC TGG GTT GAT TCT CTC AG
*cacna1c*	TAC TGC TGC TCT TCC TCT TC	ACA TCA CAG AGT TCC AGT CC
*cacna1d*	CAG GGA GAG AAG GAG TAC AA	TCA GAC TGA CCA TAG TGC TG

## Data Availability

Data is contained within the article.
